# Sex-specific association of rs16996148 SNP in the *NCAN/CILP2/PBX4 *and serum lipid levels in the Mulao and Han populations

**DOI:** 10.1186/1476-511X-10-248

**Published:** 2011-12-31

**Authors:** Ting-Ting Yan, Rui-Xing Yin, Qing Li, Ping Huang, Xiao-Na Zeng, Ke-Ke Huang, Lynn Htet Htet Aung, Dong-Feng Wu, Cheng-Wu Liu, Shang-Ling Pan

**Affiliations:** 1 Department of Cardiology, Institute of Cardiovascular Diseases, the First Affiliated Hospital, Guangxi Medical University, Nanning, Guangxi, People's Republic of China; 2 Department of Pathophysiology, School of Premedical Sciences, Guangxi Medical University, Nanning 530021, Guangxi, People's Republic of China

## Abstract

**Background:**

The association of rs16996148 single nucleotide polymorphism (SNP) in *NCAN/CILP2/PBX4 *and serum lipid levels is inconsistent. Furthermore, little is known about the association of rs16996148 SNP and serum lipid levels in the Chinese population. We therefore aimed to detect the association of rs16996148 SNP and several environmental factors with serum lipid levels in the Guangxi Mulao and Han populations.

**Method:**

A total of 712 subjects of Mulao nationality and 736 participants of Han nationality were randomly selected from our stratified randomized cluster samples. Genotyping of the rs16996148 SNP was performed by polymerase chain reaction and restriction fragment length polymorphism combined with gel electrophoresis, and then confirmed by direct sequencing.

**Results:**

The levels of apolipoprotein (Apo) B were higher in Mulao than in Han (*P *< 0.001). The frequencies of G and T alleles were 87.2% and 12.8% in Mulao, and 89.9% and 10.1% in Han (*P <*0.05); respectively. The frequencies of GG, GT and TT genotypes were 76.0%, 22.5% and 1.5% in Mulao, and 81.2%, 17.4% and 1.4% in Han (*P <*0.05); respectively. There were no significant differences in the genotypic and allelic frequencies between males and females in both ethnic groups. The levels of HDL-C, ApoAI, and the ratio of ApoAI to ApoB in Mulao were different between the GG and GT/TT genotypes in males but not in females (*P *< 0.01 for all), the subjects with GT/TT genotypes had higher serum levels of HDL-C, ApoAI, and the ratio of ApoAI to ApoB than the subjects with GG genotype. The levels of TC, TG, LDL-C, ApoAI, and ApoB in Han were different between the GG and GT/TT genotypes in males but not in females (*P *< 0.05-0.001), the T allele carriers had higher serum levels of TC, TG, LDL-C, ApoAI, and ApoB than the T allele noncarriers. The levels of HDL-C, ApoAI, and the ratio of ApoAI to ApoB in Mulao were correlated with the genotypes in males (*P *< 0.05-0.01) but not in females. The levels of TC, TG, HDL-C, LDL-C, ApoAI and ApoB in Han were associated with the genotypes in males (*P *< 0.05-0.001) but not in females. Serum lipid parameters were also correlated with several enviromental factors in both ethnic groups (*P *< 0.05-0.001).

**Conclusions:**

The genotypic and allelic frequencies of rs16996148 SNP and the associations of the SNP and serum lipid levels are different in the Mulao and Han populations. Sex (male)-specific association of rs16996148 SNP in the *NCAN/CILP2/PBX4 *and serum lipid levels is also observed in the both ethnic groups.

## Introduction

Coronary artery disease (CAD) is the leading cause of morbidity and mortality in industrialized countries, and the prevalence of this disease is increasing rapidly in developing countries [1]. Consistent and compelling evidence has demonstrated association between dyslipidemia and CAD incidence worldwide [2-4]. It is well-established that dyslipidemia is a complex trait caused by multiple environmental and genetic factors [5-7] and their interactions [8,9]. Family studies suggest that in many populations, about half of the variation in serum lipid profiles is genetically determined [10,11], and it is clear that serum lipid levels are strongly influenced by the genetic constitution of each individual.

Recent genome-wide association studies (GWAS) in different populations have identified more than 95 loci associated with serum lipid levels [5,12-46]. Common variants at these loci together explain < 10% of variation in each lipid trait [23,24,40]. Rare variants with large individual effects may also contribute to the heritability of lipid traits [40]. In addition, GWAS also discovered a number of novel loci that influence serum lipid phenotypes [15,17,21,39]. One of these newly identified single nucleotide polymorphisms (SNPs) is rs16996148 SNP in the *NCAN/CILP2/PBX4*. rs16996148 is located on chromosome 19p13 in an intergenic region between *CILP2 *and *PBX4*, which encodes a cartilage intermediate layer protein and a putative transcription factor expressed in testis, respectively [47,48]. Neurocan (NCAN) is a nervous system-specific proteoglycan involved in neuronal pattern formation, remodeling of neuronal networks and regulation of synaptic plasticity [49], with no obvious relation to LDL-C or TG concentrations. The roles of *CILP2 *and *PBX4 *in lipid metabolism are also unclear at this time [50,51]. In Europeans, however, rs16996148 in *NCAN/CILP2/PBX4 *has been showed significant associations with LDL-C and TG concentrations [15,17]. Tai et al. [51] also reported that rs16996148 SNP was significantly associated with LDL-C and HDL-C concentrations in the Malays under a recessive model of inheritance. Nevertheless, Nakayama et al. [50] did not observe significant associations between rs16996148 SNP and blood lipid profiles in the Japanese population. These results suggest that the ability of associations to generalize across other racial/ethnic populations varied greatly, some of these GWAS-identified variants may not be functional and are more likely to be in linkage disequilibrium with the functional variants.

China is a multiethnic country with 56 ethnic groups. Han nationality is the largest ethnic group, and Mulao nationality is one of the 55 minorities with population of 207,352 according to the fifth national census statistics of China in 2000. Ninety percent of them live in the Luocheng Mulao Autonomous County, Guangxi Zhuang Autonomous Region, People's Republic of China. The history of this minority can be traced back to the Jin Dynasty (AD 265-420). In a previous study, Xu et al. [52] showed that the genetic relationship between Mulao nationality and other minorities in Guangxi was much closer than that between Mulao and Han or Uighur nationality. To the best of our knowledge, however, the association of rs16996148 SNP and serum lipid levels has not been previously reported in the Chinese population. Therefore, the aim of the present study was to detect the association of rs16996148 SNP in the *NCAN/CILP2/PBX4 *and several environmental factors with serum lipid profiles in the Mulao and Han populations.

## Materials and Methods

### Participants

Participants in the present study included 712 individuals of Mulao nationality living in Luocheng Mulao Autonomous County, Guangxi Zhuang Autonomous Region, People's Republic of China. They were randomly selected from our previous stratified randomized cluster samples [53]. The ages of the participants ranged from 15 to 86 years, with an average age of 51.81 ± 14.76 years. There were 330 males (46.3%) and 382 females (53.7%). All participants were rural agricultural workers. During the same period, a total of 736 people of Han nationality who reside in the same villages were also randomly selected from our previous stratified randomized cluster samples. The average age of the subjects was 51.77 ± 14.96 years (range 15 to 86). There were 308 men (41.8%) and 428 women (58.2%). All of them were also rural agricultural workers. All study subjects were essentially healthy and had no evidence of any chronic illness, including hepatic, renal, or thyroid. The participants with a history of heart attack or myocardial infarction, stroke, congestive heart failure, diabetes or fasting blood glucose ≥ 7.0 mmol/L determined by glucose meter were excluded from the analyses. The participants were not taking medications known to affect serum lipid levels (lipid-lowering drugs such as statins or fibrates, beta-blockers, diuretics, or hormones). The experimental design was approved by the Ethics Committee of the First Affiliated Hospital, Guangxi Medical University. All participants in this study provided written informed consent.

### Epidemiological survey

The survey was carried out using internationally standardized methods [54]. All participants underwent a complete history, physical examination, and laboratory assessment of cardiovascular risk factors, including cigarette smoking, family history of myocardial infarction, blood pressure, presence of diabetes mellitus. Information on demographics, socioeconomic status, and lifestyle factors was collected with standardized questionnaires. The alcohol information included questions about the number of liangs (about 50 g) of rice wine, corn wine, rum, beer, or liquor consumed during the preceding 12 months. Alcohol consumption was categorized into groups of grams of alcohol per day: ≤ 25 and > 25. Smoking status was categorized into groups of cigarettes per day: ≤ 20 and > 20. At the physical examination, several parameters were measured. Sitting blood pressure was measured three times with the use of a mercury sphygmomanometer after the subjects had a 5-minute rest, and the average of the three measurements was used for the level of blood pressure. Systolic blood pressure was determined by the first Korotkoff sound, and diastolic blood pressure by the fifth Korotkoff sound. Body weight, to the nearest 50 grams, was measured using a portable balance scale. Subjects were weighed without shoes and in a minimum of clothing. Height was measured, to the nearest 0.5 cm, using a portable steel measuring device. From these two measurements body mass index (BMI, kg/m^2^) was calculated.

### Determination of serum lipid levels

Venous blood samples were collected after an overnight (at least 12 hours) fast. A part of the sample (2 mL) was collected into glass tubes and allowed to clot at room temperature, and used to determine serum lipid levels. Another part of the sample (3 mL) was transferred to tubes with anticoagulate solution (4.80 g/L citric acid, 14.70 g/L glucose, and 13.20 g/L tri-sodium citrate) and used to extract DNA. Serum TC, TG, HDL-C, and LDL-C levels in the samples were measured according to standard enzymatic methods. Serum ApoAI and ApoB levels were detected by the immunoturbidimetric immunoassay. All determinations were performed with an autoanalyzer (Type 7170A; Hitachi Ltd., Tokyo, Japan) in the Clinical Science Experiment Center of the First Affiliated Hospital, Guangxi Medical University [6,7].

### DNA preparation and genotyping

Genomic DNA was isolated from peripheral blood leukocytes using the phenol-chloroform method [8,9]. The extracted DNA was stored at -80°C until analysis. Genotyping of the rs16996148 SNP was performed by polymerase chain reaction and restriction fragment length polymorphism (PCR-RFLP). PCR amplification was performed using 5'-CATCCAGCATTTAGAGGTGTGA-3' and 5'-CTAGGGCAAAGGAAGTGTTTC-3' (Sangon, Shanghai, People's Republic of China) as the forward and reverse primer pairs; respectively. Each amplification reaction was performed using 100 ng (2 μL) of genomic DNA in 25 μL of reaction mixture consisting of 1.0 μL of each primer (10 μmo1/L), 12.5 μL 2 × Taq PCRMasterMix (constituent: 0.1 U *Taq *polymerase/μL, 500 μM dNTP each, 20 mM Tris-HCl, pH 8.3, 100 mM KCl, 3 mM MgCl_2_, and stabilizers), and nuclease-free water 8.5 μL. After initial denaturizing at 95°C for 5 min, the reaction mixture was subjected to 33 cycles of denaturation at 95°C for 30 s, annealing at 60°C for 45 s and extension 1 min at 72°C, followed by a final 5 min extension at 72°C. After electrophoresis on a 2.0% agarose gel with 0.5 μg/mL ethidium bromide, the amplification products were visualized under ultraviolet light. Then 2.5 U of *Hin1*II restriction enzyme, 8 μL nuclease-free water and 1 μL of 10 × buffer solution were added directly to the PCR products (5 μL) and digested at 37°C overnight. After restriction enzyme digestion of the amplified DNA, genotypes were identified by electrophoresis on 2.5% agarose gel and visualized with ethidium-bromide staining ultraviolet illumination. Genotypes were scored by an experienced reader blinded to epidemiological data and serum lipid levels.

### DNA sequencing

Six samples (GG, GT and TT genotypes in two; respectively) detected by the PCR-RFLP were also confirmed by direct sequencing. The PCR products were purified by low melting point gel electrophoresis and phenol extraction, and then the DNA sequences were analyzed in Shanghai Sangon Biological Engineering Technology & Services Co., Ltd., People's Republic of China.

### Diagnostic criteria

The normal values of serum TC, TG, HDL-C, LDL-C, ApoAI, ApoB levels and the ratio of ApoAI to ApoB in our Clinical Science Experiment Center were 3.10-5.17, 0.56-1.70, 1.16-1.42, 2.70-3.10 mmol/L, 1.20-1.60, 0.80-1.05 g/L, and 1.00-2.50; respectively [53]. The individuals with TC > 5.17 mmol/L and/or TG > 1.70 mmol/L were defined as hyperlipidemic [6,7,53]. Hypertension was diagnosed according to the criteria of 1999 World Health Organization-International Society of Hypertension Guidelines for the management of hypertension [55,56]. The diagnostic criteria of overweight and obesity were according to the Cooperative Meta-analysis Group of China Obesity Task Force. Normal weight, overweight and obesity were defined as a BMI < 24, 24-28, and > 28 kg/m^2^; respectively [57].

### Statistical analysis

The statistical analyses were done with the statistical software package SPSS 13.0 (SPSS Inc., Chicago, Illinois). Quantitative variables were expressed as mean ± standard deviation (serum TG levels were presented as medians and interquartile ranges). Qualitative variables were expressed as percentages. Allele frequency was determined via direct counting, and the standard goodness-of-fit test was used to test the Hardy-Weinberg equilibrium. Difference in genotype distribution between the groups was obtained using the chi-square test. The difference in general characteristics between two ethnic groups was tested by the Student's unpaired *t*-test. The association of genotypes and serum lipid parameters was tested by analysis of covariance (ANCOVA). Age, sex, BMI, blood pressure, alcohol consumption, and cigarette smoking were included in the statistical models as covariates. Multiple linear regression analyses adjusted for age, sex, BMI, blood pressure, alcohol consumption, and cigarette smoking were also performed to assess the association of serum lipid levels with genotypes (GG = 1, GT = 2, TT = 3; or GG = 1, GT/TT = 2) and several environment factors. A *P *value of less than 0.05 was considered statistically significant.

## Results

### General characteristics and serum lipid levels

Table 1 shows the general characteristics and serum lipid levels of the study population. The levels of ApoB and the percentages of subjects who consumed alcohol were higher but the levels of BMI and diastolic blood pressure were lower in Mulao than in Han (*P *< 0.05-0.001). There were no significant differences in the levels of age, body height, weight, systolic blood pressure, pulse pressure, blood glucose, TC, TG, HDL-C, LDL-C, ApoAI; the ratio of ApoAI to ApoB; the percentages of subjects who smoked cigarettes; and the ratio of male to female between the two ethnic groups (*P *> 0.05 for all).

**Table 1:** The general characteristics and serum lipid levels in the Mulao and Han populations

Parameter	Mulao	Han Chinese	*t *(χ^2^)	*P*
Number	712	736	-	-
Male/female	330/382	308/428	2.974	0.090
Age (years)	51.81 ± 14.76	51.77 ± 14.96	0.058	0.954
Height (cm)	155.44 ± 7.72	154.71 ± 7.93	1.774	0.076
Weight (kg)	52.88 ± 9.03	53.72 ± 8.85	-1.803	0.072
Body mass index (kg/m^2^)	21.83 ± 3.00	22.43 ± 3.32	-3.600	0.000
Systolic blood pressure (mmHg)	129.16 ± 21.47	129.58 ± 18.67	-0.397	0.692
Diastolic blood pressure (mmHg)	80.90 ± 11.60	82.27 ± 11.05	-2.298	0.022
Pulse pressure (mmHg)	48.25 ± 16.07	47.30 ± 14.19	1.187	0.235
Blood glucose	5.96 ± 1.54	5.96 ± 1.61	-0.096	0.923
Cigarette smoking [n (%)]				
Nonsmoker	519 (72.9)	532 (72.3)		
≤ 20 cigarettes/day	159 (22.3)	178 (24.2)		
> 20 cigarettes/day	34 (5.8)	26 (3.5)	1.901	0.386
Alcohol consumption [n (%)]				
Nondrinker	516 (72.5)	565 (76.8)		
≤ 25 g/day	67 (9.4)	79 (10.7)		
> 25 g/day	129 (18.1)	92 (12.5)	9.007	0.011
Total cholesterol (mmol/L)	5.02 ± 1.29	5.02 ± 1.03	0.040	0.968
Triglyceride (mmol/L)	1.05 (0.81)	1.07 (0.91)	-0.562	0.574
HDL-C (mmol/L)	1.75 ± 0.46	1.75 ± 0.53	-0.171	0.864
LDL-C (mmol/L)	2.92 ± 0.88	2.91 ± 0.85	0.337	0.736
Apolipoprotein (Apo) AI (g/L)	1.33 ± 0.40	1.35 ± 0.26	-1.456	0.146
ApoB (g/L)	0.97 ± 0.54	0.86 ± 0.20	5.202	0.000
ApoAI/ApoB	1.61 ± 0.95	1.66 ± 0.51	-1.409	0.159

HDL-C, high-density lipoprotein cholesterol; LDL-C, low-density lipoprotein cholesterol. The value of Triglyceride was presented as median (interquartile range), the difference between the two ethnic groups was determined by the Wilcoxon-Mann-Whitney test.

### Electrophoresis and genotypes

**Figure 1:**
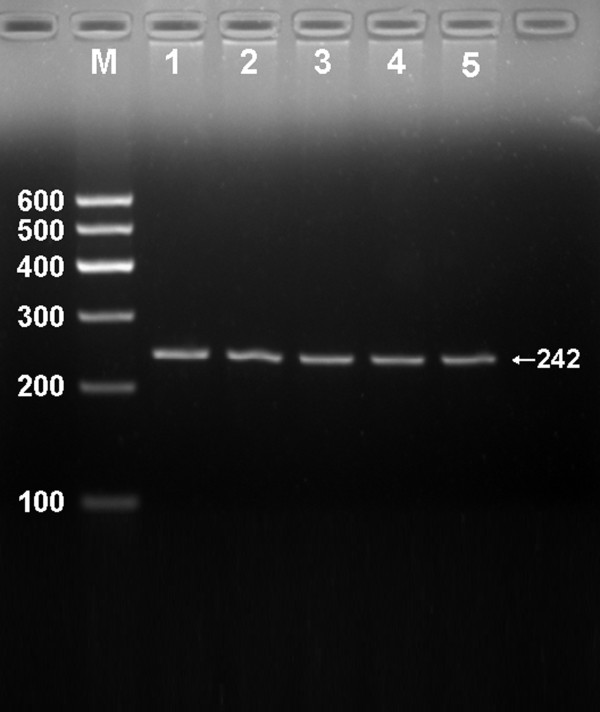
**Electrophoresis of PCR products of the samples**. Lane M, 100 bp marker ladder; lanes 1-5, samples. The 242 bp bands are the target genes.

**Figure 2:**
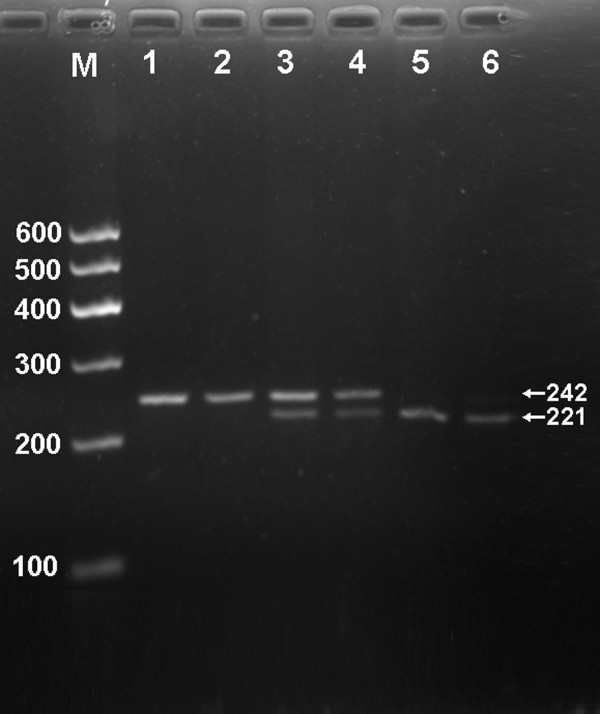
**Genotyping of rs16996148 polymorphism in the NCAN gene**. Lane M, 100 bp marker ladder; lanes 1 and 2, GG genotype (242 bp); lanes 3 and 4, GT genotype (242-, 221- and 21-bp); and lanes 5 and 6, TT genotype (221- and 21-bp). The 21 bp fragments were invisible in the gel owing to its fast migration speed.

After the genomic DNA of the samples was amplified by PCR and imaged by 2.0% agarose gel electrophoresis, the PCR products of 242 bp nucleotide sequences could be seen in the samples (Figure 1). The genotypes identified were named according to the presence (T allele) or absence (G allele) of the enzyme restriction sites. Thus, GG genotype is heterozygote for the absence of the site (bands at 242 bp), GT genotype is heterozygote for the absence and presence of the site (bands at 242-, 221- and 21-bp), and TT genotype is homozygote for the presence of the site (bands at 221- and 21- bp; Figure 2). The 21 bp fragments were invisible in the gel owing to its fast migration speed. The genotype distribution of rs16996148 SNP followed the Hardy-Weinberg equilibrium.

### Nucleotide sequences

**Figure 3:**
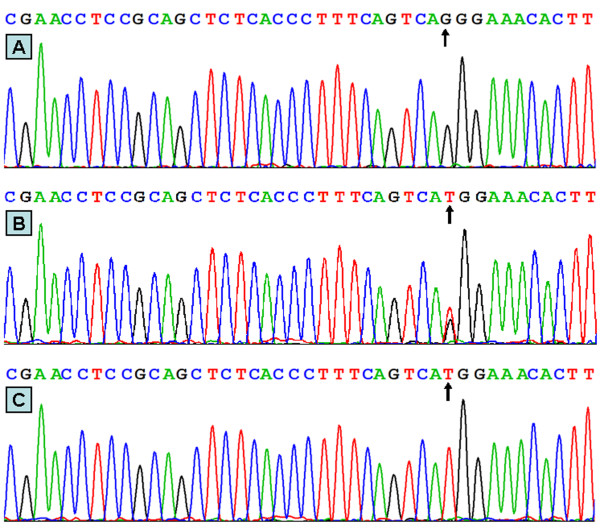
**A part of the nucleotide sequence of rs16996148 SNP**. (A) GG genotype; (B) GT genotype; (C) TT genotype.

The results were shown as GG, GT and TT genotypes of the rs16996148 SNP by PCR-RFLP, the genotypes were also confirmed by sequencing (Figure 3); respectively.

### Genotypic and allelic frequencies

The genotypic and allelic frequencies of rs16996148 SNP in the both ethnic groups are shown in Table 2. The G and T allele frequencies of rs16996148 SNP were 87.2% and 12.8% in Mulao, and 89.9% and 10.1% in Han (*P <*0.05); respectively. The frequencies of GG, GT and TT genotypes were 76.0%, 22.5% and 1.5% in Mulao, and 81.2%, 17.4% and 1.4% in Han (*P <*0.05); respectively. There were no significant differences in the genotypic and allelic frequencies between males and females in both groups.

**Table 2:** The genotypic and allelic frequencies of rs16996148 SNP the Mulao and Han populations [n (%)]

Group	n	Genotype			Allele	
			
		GG	GT	TT	G	T
Mulao	712	541 (76.0)	160 (22.5)	11 (1.5)	1242 (87.2)	182 (12.8)
Han	736	598 (81.2)	128 (17.4)	10 (1.4)	1324 (89.9)	148 (10.1)
χ^2^	-		6.060		5.329	
*P*	-		0.048		0.022	
Mulao						
Male	330	243 (73.6)	79 (24.0)	8 (2.4)	565 (85.6)	95 (14.4)
Female	382	298 (78.0)	81 (21.2)	3 (0.8)	677 (88.6)	87 (11.4)
χ^2^	-		4.113		2.871	
*P*	-		0.128		0.095	
Han						
Male	308	246 (79.9)	60 (19.5)	2 (0.6)	552 (89.6)	64 (10.4)
Female	428	352 (82.2)	68 (15.9)	8 (1.9)	772 (90.2)	84 (9.8)
χ^2^	-		3.415		0.132	
*P*	-		0.181		0.726	

### Genotypes and serum lipid levels

As shown in Table 3, the levels of HDL-C in Mulao were different among the three genotypes (*P *< 0.05), the T allele carriers had higher serum HDL-C levels than the T allele noncarriers. When serum lipid parameters in Mulao were analyzed according to sex, we found that the levels of HDL-C, ApoAI, and the ratio of ApoAI to ApoB in males but not in females were different between the GG and GT/TT genotypes (*P *< 0.01 for all), the subjects with GT/TT genotypes had higher serum levels of HDL-C, ApoAI, and the ratio of ApoAI to ApoB than the subjects with GG genotype.

**Table 3:** The genotypes of rs16996148 SNP and serum lipid levels in the Mulao and Han populations

Genotype	n	TC (mmol/L)	TG (mmol/L)	HDL-C (mmol/L)	LDL-C (mmol/L)	ApoAI (g/L)	ApoB (g/L)	ApoAI/ ApoB
Mulao								
GG	541	5.03 ± 1.35	1.07(0.84)	1.72 ± 0.44	2.94 ± 0.88	1.31 ± 0.40	0.98 ± 0.56	1.59 ± 1.01
GT	160	4.99 ± 1.11	1.02(0.73)	1.83 ± 0.50	2.88 ± 0.91	1.37 ± 0.41	0.94 ± 0.49	1.68 ± 0.73
TT	11	4.91 ± 0.85	1.18(0.89)	1.89 ± 0.20	2.78 ± 0.74	1.32 ± 0.47	0.93 ± 0.46	1.54 ± 0.62
*F*	-	0.108	1.338	3.910	0.284	1.593	0.564	0.685
*P*	-	0.897	0.512	0.020	0.752	0.204	0.569	0.505
GG	541	5.03 ± 1.35	1.07(0.84)	1.72 ± 0.44	2.94 ± 0.88	1.31 ± 0.40	0.98 ± 0.56	1.59 ± 1.01
GT/TT	171	4.99 ± 1.09	1.03(0.74)	1.83 ± 0.49	2.87 ± 0.90	1.37 ± 0.41	0.94 ± 0.49	1.67 ± 0.73
*F*	-	0.045	-1.154	7.483	0.398	2.981	1.028	1.304
*P*	-	0.832	0.248	0.006	0.528	0.085	0.311	0.254
Han								
GG	598	4.95 ± 0,98	1.07(0.86)	1.74 ± 0.55	2.86 ± 0.81	1.35 ± 0.26	0.85 ± 0.20	1.67 ± 0.52
GT	128	5.28 ± 1.16	1.15(1.23)	1.76 ± 0.44	3.13 ± 0.94	1.37 ± 0.23	0.90 ± 0.21	1.59 ± 0.43
TT	10	5.40 ± 1.38	0.84(0.49)	2.17 ± 0.41	3.05 ± 1.26	1.62 ± 0.33	0.83 ± 0.26	2.15 ± 0.79
*F*		7.467	6.323	2.473	5.504	5.845	2.909	1.940
*P*		0.001	0.042	0.085	0.004	0.003	0.055	0.145
GG	598	4.95 ± 0.98	1.07(0.86)	1.74 ± 0.55	2.86 ± 0.81	1.35 ± 0.26	0.85 ± 0.20	1.67 ± 0.52
GT/TT	138	5.29 ± 1.17	1.15(1.18)	1.79 ± 0.45	3.12 ± 0.96	1.38 ± 0.25	0.89 ± 0.21	1.63 ± 0.49
*F*	-	12.684	-1.555	1.389	10.222	4.955	5.469	0.091
*P*	-	0.000	0.120	0.239	0.001	0.026	0.020	0.763
Mulao/male								
GG	243	5.18 ± 1.50	1.15(1.07)	1.72 ± 0.45	2.96 ± 0.86	1.32 ± 0.44	1.04 ± 0.62	1.48 ± 0.65
GT/TT	87	5.08 ± 1.64	1.08(0.86)	1.86 ± 0.58	2.84 ± 0.86	1.44 ± 0.40	0.98 ± 0.52	1.70 ± 0.77
*F*	-	0.276	-0.341	7.151	1.375	7.126	0.574	9.195
*P*	-	0.600	0.733	0.008	0.245	0.008	0.449	0.003
Mulao/female								
GG	298	4.91 ± 1.21	1.03(0.65)	1.72 ± 0.44	2.92 ± 0.89	1.31 ± 0.37	0.93 ± 0.50	1.68 ± 1.22
GT/TT	84	4.89 ± 1.01	0.95(0.60)	1.80 ± 0.38	2.90 ± 0.95	1.29 ± 0.41	0.89 ± 0.46	1.63 ± 0.68
*F*	-	0.110	-1.648	1.134	0.181	0.104	0.106	0.163
*P*	-	0.740	0.099	0.288	0.671	0.747	0.745	0.686
Han/male								
GG	246	5.08 ± 0.90	1.14(0.92)	1.69 ± 0.42	2.90 ± 0.77	1.36 ± 0.28	0.90 ± 0.19	1.58 ± 0.47
GT/TT	62	5.59 ± 1.12	1.71(1.61)	1.75 ± 0.45	3.26 ± 0.78	1.44 ± 0.28	0.97 ± 0.23	1.56 ± 0.46
*F*	-	13.935	-2.649	2.569	8.390	7.843	4.675	0.248
*P*	-	0.000	0.008	0.110	0.004	0.005	0.031	0.619
Han/female								
GG	352	4.86 ± 1.02	1.00(0.81)	1.78 ± 0.62	2.83 ± 0.84	1.33 ± 0.24	0.81 ± 0.20	1.74 ± 0.54
GT/TT	76	5.05 ± 1.16	0.93(0.80)	1.81 ± 0.46	3.01 ± 1.08	1.34 ± 0.21	0.84 ± 0.18	1.69 ± 0.50
*F*	-	3.262	-0.389	0.133	3.680	0.141	1.239	0.548
*P*	-	0.072	0.698	0.715	0.056	0.707	0.266	0.459

TC, total cholesterol; TG, triglyceride; HDL-C, high-density lipoprotein cholesterol; LDL-C, low-density lipoprotein cholesterol; ApoAI, apolipoprotein AI; ApoB, apolipoprotein B

In the Han population, the levels of TC, TG, LDL-C and ApoAI among the three genotypes, or the levels of TC, LDL-C, ApoAI, and ApoB between the GG and GT/TT genotypes were different (*P *< 0.05-0.001), the T allele carriers had higher serum TC, TG, LDL-C, ApoAI and ApoB levels than the T allele noncarriers. When serum lipid parameters in Han were stratified according to sex, we showed that the levels of TC, TG, LDL-C, ApoAI, and ApoB in males but not in females were different between the GG and GT/TT genotypes (*P *< 0.05-0.001), the subjects with GT/TT genotypes had higher serum levels of TC, TG, LDL-C, ApoAI, and ApoB than the subjects with GG genotype.

### Risk factors for serum lipid parameters

The correlation between the genotypes of rs16996148 SNP and serum lipid parameters in Mulao and Han is shown in Table 4. The levels of HDL-C, ApoAI, and the ratio of ApoAI to ApoB in Mulao were correlated with the genotypes in males (*P *< 0.05-0.01) but not in females. The levels of TC, TG, HDL-C, LDL-C, ApoAI and ApoB in Han were associated with the genotypes in males (*P *< 0.05-0.001) but not in females.

**Table 4:** Correlation between the genotypes of rs16996148 SNP and serum lipid parameters in the Mulao and Han populations

Lipid parameter	Relative factor	Unstandardized coefficient	Std. error	Standardized coefficient	*t*	*P*
Mulao plus Han						
TC	Genetype	0.137	0.065	0.053	2.106	0.035
HDL-C	Genetype	0.082	0.028	0.075	2.924	0.004
ApoAI	Genetype	0.049	0.019	0.066	2.577	0.010
Mulao						
HDL-C	Genetype	0.091	0.035	0.094	2.609	0.009
Han						
TC	Genetype	0.322	0.082	0.136	3.934	0.000
TG	Genetype	0.335	0.161	0.074	2.082	0.038
LDL-C	Genetype	0.234	0.068	0.119	3.424	0.001
ApoAI	Genetype	0.057	0.020	0.096	2.802	0.005
ApoB	Genetype	0.038	0.015	0.081	2.482	0.013
Mulao/male						
HDL-C	Genetype	0.133	0.050	0.137	2.647	0.009
ApoAI	Genetype	0.109	0.045	0.128	2.419	0.016
ApoAI/ApoB	Genetype	0.192	0.072	0.141	2.676	0.008
Han/male						
TC	Genetype	0.513	0.122	0.223	4.203	0.000
TG	Genetype	0.860	0.345	0.139	2.493	0.013
HDL-C	Genetype	0.114	0.053	0.113	2.159	0.032
LDL-C	Genetype	0.327	0.101	0.176	3.241	0.001
ApoAI	Genetype	0.107	0.033	0.159	3.268	0.001
ApoB	Genetype	0.058	0.024	0.121	2.388	0.018

Serum lipid parameters were also correlated with several environment factors such as age, gender, alcohol consumption, cigarette smoking, blood pressure, blood glucose, and BMI in both ethnic groups (*P *< 0.05-0.001; Table 5).

**Table 5:** Correlation between environmental factors and serum lipid parameters in the Mulao and Han populations

Lipid parameter	Relative factor	Unstandardized coefficient	Std. error	Standardized coefficient	*t*	*P*
Mulao plus Han						
TC	Body mass index	0.053	0.010	0.145	5.530	0.000
	Age	0.012	0.002	0.155	5.960	0.000
	Alcohol consumption	0.177	0.040	0.113	4.379	0.000
	Diastolic blood pressure	0.006	0.003	0.054	2.004	0.045
TG	Body mass index	0.107	0.016	0.170	6.582	0.000
	Gender	-0.309	0.127	-0.077	-2.445	0.015
	Alcohol consumption	0.214	0.085	0.079	2.509	0.012
	Blood glucose	0.080	0.032	0.063	2.464	0.014
HDL-C	Body mass index	-0.030	0.004	-0.191	-7.412	0.000
	Alcohol consumption	0.105	0.021	0.157	4.981	0.000
	Gender	0.105	0.031	0.105	3.348	0.001
	Age	0.002	0.001	0.058	2.261	0.024
LDL-C	Body mass index	0.051	0.007	0.186	7.334	0.000
	Age	0.010	0.002	0.174	6.673	0.000
	Blood glucose	0.030	0.014	0.054	2.082	0.038
ApoAI	Alcohol consumption	0.131	0.014	0.287	9.143	0.000
	Body mass index	-0.010	0.003	-0.090	-3.483	0.001
	Age	0.002	0.001	0.073	2.863	0.004
	Gender	0.061	0.021	0.089	2.850	0.004
	Nation	-0.045	0.017	-0.066	-2.586	0.010
ApoB	Body mass index	0.021	0.003	0.165	6.425	0.000
	Nation	0.121	0.021	0.147	5.768	0.000
	Blood glucose	0.024	0.007	0.092	3.611	0.000
	Gender	-0.075	0.021	-0.091	-3.537	0.000
	Pulse pressure	0.001	0.001	0.054	2.109	0.035
ApoAI/ApoB	Body mass index	-0.047	0.006	-0.197	-7.634	0.000
	Blood glucose	-0.036	0.013	-0.076	-2.904	0.004
	Gender	0.207	0.048	0.135	4.320	0.000
	Alcohol consumption	0.118	0.032	0.115	3.663	0.000
	Age	-0.003	0.001	-0.066	-2.526	0.012
	Nation	-0.087	0.039	-0.058	-2.247	0.025
Mulao						
TC	Body mass index	0.066	0.016	0.153	4.163	0.000
	Age	0.011	0.003	0.126	3.455	0.001
	Alcohol consumption	0.179	0.061	0.108	2.953	0.003
TG	Body mass index	0.126	0.025	0.186	5.088	0.000
	Alcohol consumption	0.407	0.095	0.156	4.275	0.000
HDL-C	Body mass index	-0.037	0.006	-0.244	-6.727	0.000
	Alcohol consumption	0.066	0.021	0.113	3.115	0.002
LDL-C	Body mass index	0.054	0.011	0.182	4.997	0.000
	Age	0.009	0.002	0.146	3.998	0.000
ApoAI	Alcohol consumption	0.095	0.019	0.184	5.009	0.000
	Age	0.002	0.001	0.088	2.393	0.017
ApoB	Body mass index	0.026	0.007	0.142	3.830	0.000
	Gender	-0.087	0.040	-0.080	-2.16	0.031
ApoAI/ApoB	Body mass index	-0.041	0.012	-0.129	-3.468	0.001
	Blood glucose	-0.052	0.023	-0.084	-2.260	0.024
Han Chinese						
TC	Diastolic blood pressure	0.014	0.003	0.147	3.981	0.000
	Age	0.012	0.002	0.180	5.005	0.000
	Body mass index	0.041	0.011	0.133	3.688	0.000
	Alcohol consumption	0.188	0.052	0.127	3.610	0.000
TG	Body mass index	0.082	0.022	0.138	3.744	0.000
	Blood glucose	0.151	0.043	0.125	3.500	0.000
	Gender	-0.474	0.144	-0.119	-3.301	0.001
	Diastolic blood pressure	0.017	0.006	0.094	2.560	0.011
HDL-C	Body mass index	-0.022	0.006	-0.141	-3.811	0.000
	Alcohol consumption	0.110	0.034	0.144	3.250	0.001
	Gender	0.228	0.054	0.213	4.196	0.000
	Cigarette smoking	0.122	0.046	0.123	2.640	0.008
LDL-C	Age	0.010	0.002	0.185	4.946	0.000
	Body mass index	0.047	0.009	0.182	5.052	0.000
	Blood glucose	0.050	0.019	0.094	2.624	0.009
	Diastolic blood pressure	0.006	0.003	0.074	1.992	0.047
ApoAI	Alcohol consumption	0.129	0.016	0.345	8.253	0.000
	Body mass index	-0.014	0.003	-0.175	-4.997	0.000
	Cigarette smoking	0.088	0.021	0.182	4.119	0.000
	Gender	0.099	0.025	0.189	3.942	0.000
ApoB	Body mass index	0.018	0.002	0.300	8.877	0.000
	Blood glucose	0.023	0.004	0.182	5.433	0.000
	Gender	-0.046	0.016	-0.112	-2.819	0.005
	Systolic blood pressure	0.001	0.000	0.093	2.446	0.015
	Age	0.001	0.001	0.104	2.802	0.005
	Alcohol consumption	0.026	0.012	0.089	2.230	0.026
ApoAI/ApoB	Body mass index	-0.050	0.005	-0.323	-9.364	0.000
	Age	-0.004	0.001	-0.112	-3.234	0.001
	Blood glucose	-0.026	0.011	-0.082	-2.347	0.019
	Gender	0.273	0.049	0.264	5.575	0.000
	Cigarette smoking	0.154	0.042	0.161	3.683	0.000
	Alcohol consumption	0.098	0.031	0.133	3.225	0.001

TC, total cholesterol; TG, triglyceride; HDL-C, high-density lipoprotein cholesterol; LDL-C, low-density lipoprotein cholesterol; ApoAI, apolipoprotein AI; ApoB, apolipoprotein B

## Discussion

The present study shows that serum ApoB levels were higher in Mulao than in Han nationalities. There were no significant differences in the remaining serum lipid parameters between the two ethnic groups. It is well known that dyslipidemia is the result of a combination of genetic and environmental factors [6-9]. Both family and twin studies suggest that in many populations, about 40-60% of the variation in serum lipid profiles is genetically determined [10,11], and it is clear that LDL-C, HDL-C and TG concentrations are strongly influenced by the genetic constitution of each individual. The engagements of Mulao nationality were family-arranged in childhood, usually with the girl being four or five years older than the boy. There was a preference for marriage to mother's brother's daughter. Engagement and marriage were marked by bride-wealth payments. Marriage ceremonies were held when the girl reached puberty. She remained with her natal family until her first child was born. Till then she was free to join the young men and women who came together for responsive singing, flirtations, and courtships at festival times. Divorce and remarriage were permitted, with little restriction. The two-generation household is the most common unit of residence. Households are under the control of the father, and divide when the sons marry, with only the youngest son remaining with the parents. Therefore, we believe that the genetic background and some lipid-associated genetic variants in this population may be different from those in Han nationality.

The genotypic and allelic frequencies of rs16996148 SNP in the *NCAN/CILP2/PBX4 *in diverse racial/ethnic groups are inconsistent. The frequency of T allele was 8% in European Americans, 15% in African Americans, 4% in American Indians, 6% in Mexican Americans and Hispanics [40], and 12% in Japanese [50]. The minor allele frequency in the Malay population was 17% [51]. In the present study, we showed that the T allele frequency of rs16996148 SNP was higher in Mulao than in Han (12.8% vs. 10.1%, *P <*0.05). The frequencies of GG, GT and TT genotypes were also different between the two ethnic groups (*P <*0.05). There were no significant differences in the genotypic and allelic frequencies between males and females in both ethnic groups. These results indicate that the prevalence of the T allele variant of the rs16996148 SNP may have a racial/ethnic specificity.

The potential relationship between the rs16996148 SNP and plasma or serum lipid levels in humans has been evaluated in several previous studies (GWAS). However, previous findings on the association of this SNP with the changes in plasma lipid levels are inconsistent. The rs16996148 SNP in *NCAN/CILP2/PBX4 *has been shown significant associations with LDL-C and TG concentrations in Europeans [15,17]. The minor allele (T allele) of rs16996148 SNP was associated with lower concentrations of both LDL-C (by ~16 mg/dl) and TG [15]. Tai et al. [51] also reported that rs16996148 SNP was significantly associated with lower LDL-C and elevated HDL-C concentrations in the Malays under a recessive model of inheritance [51]. Nevertheless, Nakayama et al. [50] did not observe significant associations between rs16996148 and blood lipid profiles in the Japanese population. In the current study, we found that the levels of HDL-C, ApoAI, and the ratio of ApoAI to ApoB in Mulao were different between the GG and GT/TT genotypes in males but not in females, the subjects with GT/TT genotypes had higher serum levels of HDL-C, ApoAI, and the ratio of ApoAI to ApoB than the subjects with GG genotype. The levels of TC, TG, LDL-C, ApoAI, and ApoB in Han were different between the GG and GT/TT genotypes in males but not in females, the T allele carriers had higher serum levels of TC, TG, LDL-C, ApoAI, and ApoB than the T allele noncarriers. The levels of HDL-C, ApoAI, and the ratio of ApoAI to ApoB in Mulao were correlated with the genotypes in males but not in females. The levels of TC, TG, HDL-C, LDL-C, ApoAI and ApoB in Han were correlated with the genotypes in males but not in females. These findings suggest that there is a sex-specific association of rs16996148 SNP in the *NCAN/CILP2/PBX4 *and serum lipid levels in our study populations.

It is well known that environmental factors such as dietary patterns, lifestyle, obesity, physical activity, and hypertension are all strongly related with serum lipid levels [6,7]. Furthermore, exposure to different lifestyles and environments in our populations resident in Guangxi may further modify the effect of genetic variation on blood lipids. In the present study, we also showed that serum lipid parameters were correlated with age, sex, alcohol consumption, cigarette smoking, BMI, and blood pressure in both ethnic groups. These data suggest that the environmental factors also play an important role in determining serum lipid levels in our populations. Although rice and corn are the staple foods in both ethnic groups, the people of Mulao nationality like to eat cold foods along with acidic and spicy dishes, so bean soy sauce and pickled vegetables are among their most popular dishes. They also like to eat animal offals which contain abundant saturated fatty acid. The effects of dietary macronutrients on serum lipid levels and their effects on CAD have been extensively studied [58-62]. Almost 40 y ago, the Puerto Rican Heart Study found lower mean concentrations of TC and TG in Puerto Ricans than in subjects in the Framingham Heart Study [63]. Among urban Puerto Rican men, TC was positively associated with the percentage of energy from total fat, saturated fatty acids (SFAs), simple sugars, and protein and negatively associated with the percentage of energy from polyunsaturated fatty acids (PUFAs), total carbohydrate, and PUFA/SFA. Overall, diet and relative weight can account for at most 6% of the variability in serum cholesterol observed, with at most 2.5% of the variability due diet alone [63].

## Conclusion

The present study shows that the T allele frequency of rs16996148 SNP in the *NCAN/CILP2/PBX4 *is significantly higher in Mulao than in Han. The frequencies of GG, GT and TT genotypes are also different between the two ethnic groups. The subjects with GT/TT genotypes in Mulao had higher serum levels of HDL-C, ApoAI, and the ratio of ApoAI to ApoB than the subjects with GG genotype in males but not in females. The T allele carriers in Han had higher serum levels of TC, TG, LDL-C, ApoAI, and ApoB than the T allele noncarriers in males but not in females. These lipid parameters were also correlated with the genotypes in males but not in females. These results suggest that there is a sex-specific association of rs16996148 SNP in the *NCAN/CILP2/PBX4 *and serum lipid levels in our study populations.

## Competing interests

The authors declare that they have no competing interests.

## Authors' contributions

TTY participated in the design, undertook genotyping, and helped to draft the manuscript. RXY conceived the study, participated in the design, carried out the epidemiological survey, collected the samples, and drafted the manuscript. QL, PH, XNZ, KKH, LHHA and DFW carried out the epidemiological survey and collaborated to the genotyping. CWL and SLP carried out the epidemiological survey and collected the samples. All authors read and approved the final manuscript.
